# Surrogate Perspectives on Patient Preference Predictors: Good Idea, but I Should Decide How They Are Used

**DOI:** 10.1080/23294515.2022.2040643

**Published:** 2022-03-08

**Authors:** Dana Howard, Allan Rivlin, Philip Candilis, Neal W. Dickert, Claire Drolen, Benjamin Krohmal, Mark Pavlick, David Wendler

**Affiliations:** a Center for Bioethics, Ohio State University, Columbus, OH, USA; b Zen Political Research, Washington, DC, USA; c Saint Elizabeths Hospital, Washington, DC, USA; d Medicine, Emory University, Atlanta, GA, USA; e UCLA Medical School, Los Angeles, CA, USA; f John J. Lynch MD Center for Ethics, MedStar Washington Hospital Center, Washington, DC, USA; g Emergency Medicine, Georgetown University School of Medicine, Washington, DC, USA; h St Elizabeths Hospital, Washington, DC, USA; i Department of Bioethics, Clinical Center, National Institutes of Health, Bethesda, USA

**Keywords:** Predictive algorithms, surrogate decision-making, critical care ethics, advance care planning

## Abstract

**Background:**

Current practice frequently fails to provide care consistent with the preferences of decisionally-incapacitated patients. It also imposes significant emotional burden on their surrogates. Algorithmic-based patient preference predictors (PPPs) have been proposed as a possible way to address these two concerns. While previous research found that patients strongly support the use of PPPs, the views of surrogates are unknown. The present study thus assessed the views of experienced surrogates regarding the possible use of PPPs as a means to help make treatment decisions for decisionally-incapacitated patients.

**Methods:**

This qualitative study used semi-structured interviews to determine the views of experienced surrogates [*n* = 26] who were identified from two academic medical centers and two community hospitals. The primary outcomes were respondents’ overall level of support for the idea of using PPPs and the themes related to their views on how a PPP should be used, if at all, in practice.

**Results:**

Overall, 21 participants supported the idea of using PPPs. The remaining five indicated that they would not use a PPP because they made decisions based on the patient’s best interests, not based on substituted judgment. Major themes which emerged were that surrogates, not the patient’s preferences, should determine how treatment decisions are made, and concern that PPPs might be used to deny expensive care or be biased against minority groups.

**Conclusions:**

Surrogates, like patients, strongly support the idea of using PPPs to help make treatment decisions for decisionally-incapacitated patients. These findings provide support for developing a PPP and assessing it in practice. At the same time, patients and surrogates disagree over whose preferences should determine how treatment decisions are made, including whether to use a PPP. These findings reveal a fundamental disagreement regarding the guiding principles for surrogate decision-making. Future research is needed to assess this disagreement and consider ways to address it.

Many patients, especially those at the end of life, are unable to make their own treatment decisions (Raymont et al. 2004; Silveira, Kim, and Langa 2010). One study found that up to 69% of nursing home residents have decisional impairment and, 48 hours after hospitalization, 47.4% of adults 65 and older required the involvement of a surrogate decision-maker (Torke et al. 2014).

Surrogate decision-makers, whether they are designated by the patient or are the patient’s next of kin, are charged with making treatment decisions based on the patient’s expressed preferences. When the patient’s preferences are unknown, and it is unclear which decision would best promote the patient’s interests, surrogates are instructed to make decisions based on what they think the patient would have chosen in the circumstances (i.e., the substituted judgment standard). According to one of the seminal texts on decision making for incapacitated patients, surrogates should ask “if the patient miraculously were to awaken for a few moments” what treatment decision would they make (Buchanan and Brock 1989). This approach is meant to promote patient autonomy by basing treatment decisions on the preferences and values the patient endorsed, even after the patient no longer has decisional capacity (Beauchamp and Childress 2019).

Reliance on surrogates to implement the substituted judgment standard keeps the patient’s family and loved ones involved in their care. And, because families and loved ones typically know the patient well, it is assumed that this approach offers the best way to provide medical care consistent with the patient’s preferences and values. Unfortunately, current implementation of the substituted judgment standard raises two significant concerns.

First, surrogates are frequently unable to predict which treatment patients would choose for themselves. In studies using hypothetical scenarios, surrogates accurately predict whether their loved one would accept or refuse a given course of treatment 54%- 68% of the time, where random guessing would have yielded 50% accuracy (Uhlmann, Pearlman, and Cain 1988; FAMIREA Study Group, 2005). Second, because decisional incapacity frequently occurs at the end of life, many of the decisions surrogates are asked to make have profound implications for their loved ones. Moreover, some surrogates may not understand their role. A recent study found that clinicians often fail to offer guidance, or they offer conflicting guidance regarding how surrogates should make decisions. As a result, some surrogates may be ill-prepared for the high stakes decisions they find themselves needing to make (Halpern et al. 2013). Given the significance of these decisions, and the difficulty predicting which option the patient would have chosen, surrogates often experience deep and long-lasting emotional distress. One study found that a third of surrogates charged with making decisions for loved ones in intensive care units developed symptoms associated with posttraumatic stress disorder (FAMIREA Study Group, 2005). This number increased to nearly 82% for surrogates who made end-of-life decisions (French FAMIREA Study Group, 2005; Cunningham et al. 2018)

Commentators have endorsed a number of approaches to try to address these two concerns. Unfortunately, no method has been found to reliably improve surrogate accuracy or reduce surrogate burden. A prominent recommendation is to encourage patients to discuss their clinical preferences while competent with their assigned surrogate (Vig et al. 2007). However, studies find that prior conversations between patient and surrogate do not improve surrogates’ ability to predict which treatment the patient would have chosen (Bravo et al. 2016; Ditto et al. 2001). Another proposal is to provide surrogates with information about the patient’s prognosis, as well as guided exercises to help the surrogate clarify the patient’s values. This approach also has not been found to improve surrogates’ predictive accuracy (Cox et al. 2019).

Other approaches attempt to reduce surrogates’ emotional burden. One study assessed whether guideline-based strategies for providing emotional support to surrogates, and ensuring frequent clinician–family communication in the ICU, might reduce symptoms of post-traumatic stress disorder (White et al. 2018). Although the study found that the intervention improved surrogates’ rating of the clinician–family communication, it did not reduce surrogates’ emotional burden. Another study of the family members of patients with chronic critical illness found that the use of palliative care–led informational and emotional support meetings did not reduce anxiety or depression symptoms, and may have increased post-traumatic stress disorder symptoms (Carson et al. 2016).

Given the difficulties improving surrogate accuracy and reducing surrogate burden, some commentators advocate reconceptualizing substituted judgment. Rather than attempting to predict the preferences of incapacitated patients, some ethicists endorse a shared decision-making model, where the surrogate describes the patient’s interests and values and the physicians make recommendations for treatment (Sulmasy and Snyder 2010).

The primary problem with this approach is that a majority of patients prefer that their loved ones make treatment decisions for them in the event that they become incapable of doing so (Rid and Wendler 2010; Brenna, Rid, and Wendler 2012; Nolan et al. 2005; High 1990). This preference is particularly pronounced in marginalized populations, and in the US, especially within the African American community, who report higher levels of suspicion that healthcare systems limit which treatments they can receive (Perkins et al. 2002; Washington 2007; Eaton et al. 2015). In addition, physicians have been found to be even less able to predict patients’ treatment preferences than family members (Uhlmann, Pearlman, and Cain 1988; Coppola et al. 2001). Moving away from substituted judgment may therefore decrease the extent to which patients are treated consistent with their preferences and values, especially when it comes to preferences regarding the decision-making process itself. Other commentators suggest that surrogates should rely more on their own preferences as opposed to trying to predict the treatment preferences of the patient (Berger, DeRenzo, and Schwartz 2008). This approach is consistent with proposals to give surrogates greater “leeway” when making decisions for incapacitated patients (Kim et al. 2013). However, these approaches also seem inconsistent with the preferences of patients, and are unlikely to reduce surrogate burden (Brenna, Rid, and Wendler 2012; Rid et al. 2015).

It is perhaps not surprising that efforts to date have not been able to improve surrogates’ predictive accuracy nor decrease their emotional burden. And it is not surprising, in light of these challenges, that some commentators have proposed to abandon substituted judgment. Surrogates are asked to make life and death decisions for loved ones, frequently when it is unclear what is medically best for the patient. In these cases, people, such as some Jehovah Witnesses who decline blood transfusions in all circumstances, have clear and longstanding treatment preferences. In other cases, knowing a person, even for decades, may not provide insight into whether they would regard the potential benefits of treatment as outweighing the risks and burdens. Making treatment decisions for acutely ill loved ones thus often leaves surrogates feeling responsible for unwanted patient outcomes. To address these concerns, we need an approach that provides greater evidence of the patient’s treatment wishes and takes at least some of the burdens of decisional responsibility off the surrogate, without removing them from the decision-making process entirely.

Some have proposed to try to achieve these aims by supplementing current practice with a Patient Preference Predictor (PPP) (Rid and Wendler, 2014; Rid and Wendler, 2014). A PPP would be an algorithmic model that predicts which treatment the patient would want based on the preferences of similar patients, possibly supplemented with information about the patient from their electronic health records and online profile (Biller-Andorno and Biller 2019). A preliminary PPP was found to be as accurate as surrogates in predicting patient preferences (Shalowitz, Garrett-Mayer, and Wendler 2007). Because the tested PPP did not include factors known to be correlated with patients’ treatment preferences, such as their age, this finding suggests that a more comprehensive PPP, one that incorporates a broader range of predictive factors, might be more accurate than surrogates alone. Moreover, previous studies find that a good deal of surrogates’ emotional burden traces to their not knowing the patient’s treatment preferences (Rid and Wendler, 2014). Hence, use of a PPP, if it increases surrogates’ predictive accuracy, might also reduce their emotional burden.

Development and testing of a comprehensive PPP to determine whether, in fact, it is more accurate than surrogates alone would be expensive and time-consuming. Hence, before expending these resources, it is important to assess whether its use would be feasible and welcome in practice.

A prior study found, that, if use of a PPP does increase surrogates’ predictive accuracy, a majority of patients support its use (Wendler et al. 2016). However, surrogates are the ones who would be involved in implementing the PPP in practice. Hence, whether use of a PPP would be feasible and welcome depends critically on the views of surrogates. If surrogates are skeptical of such a tool, they may not make use of its predictions in their decision-making and it may lead to further stress, regardless of patient endorsement. The present manuscript thus provides the first assessment of the views of surrogates regarding two questions: Do surrogates support the idea of a PPP? How, if at all, do surrogates think a PPP should be incorporated into practice?

## Methods

### Study development

To ensure respondents understood the idea of a PPP, and to get more in-depth information about surrogates’ attitudes and experiences, we conducted semi-structured qualitative interviews. This methodology, rather than the use of forced choice, is suitable for exploring the perspectives of surrogate decision-makers when little empirical work is available on the subject. To supplement the qualitative data, two written surveys were also developed, one that was completed prior to, and the other following the interview.

The interview guide (Online Appendix Text 1, supplementary materials) and quantitative surveys (Online Appendix Text 2 and Appendix Text 3, supplementary materials) were designed after an extensive literature review. Draft versions were reviewed by three content experts on surrogate decision making, and revised accordingly. All the instruments then underwent cognitive and behavioral testing with five surrogates.

### Recruitment

To try to ensure a diverse group of respondents, participants were recruited from two university-affiliated hospitals, Emory University Hospital, Atlanta, GA, and the Hospital of the University of Pennsylvania, Philadelphia, PA, and two community hospitals, Saint Elizabeths Hospital, Washington, DC, a government-run facility for individuals with severe persistent mental illness, and Medstar Washington Hospital Center, Washington, DC. We used convenience sampling, with clinicians who knew eligible surrogates briefly explaining the study and putting those who expressed interest in touch with the moderator to schedule an interview. To ensure participants were familiar with surrogate decision-making, we enrolled only ‘experienced’ surrogates, defined as surrogates who had made at least one medical decision for an incapacitated adult within the past three years.

### Interview process

The interviews were conducted in person or by phone. All the sessions were conducted by the same moderator (AR) who is an expert in qualitative methods and has no connection with the development of the PPP. All sessions were recorded and transcribed verbatim.

The goal of the interviews was to assess experienced surrogates’ views of the idea of a PPP and their preferences for how such a tool, if it is developed, should be used, if at all. Just prior to the interview, participants filled out a pre-interview questionnaire [Online Appendix Text 2, supplementary materials] that solicited information about their relationship to the patient and some general characteristics of their surrogate decision-making, their views of the role surrogates should play in decision-making, and their level of confidence regarding the correctness of the choices they made on behalf of the patient. The moderator opened the interview by asking participants to describe their experience making treatment decisions for an incapacitated patient and, for the purposes of the interview, to focus on one decision, either the most recent or the most memorable one. The moderator described the idea of the PPP in detail, explained that researchers were considering whether it makes sense to develop a comprehensive PPP, and answered any clarificatory questions. The moderator then solicited participants’ overall views of the idea of a PPP. Regardless of their overall level of support, all participants were asked what they saw as the advantages and disadvantages of using a PPP were it to be developed.

Handouts (Online Appendix Text 4, 5, 6, and 7, supplementary materials) were used at several points to facilitate participant understanding. Some of the questions in the handouts involved numerical ratings of ideas and multiple-choice questions. These activities were meant to guard against ‘group effect’ in interviews involving more than one participant and were a way for individuals to anchor their perspective prior to hearing the attitudes of others. Finally, the interview ended with participants filling out a post-interview questionnaire to solicit their views on whether PPPs should be offered, whether they would have used such a tool if offered, and how it would have affected their decision-making. For phone interviews, participants were mailed all handouts and questionnaires ahead of time, along with a postmarked return envelope. Respondents filled out the forms as instructed by the moderator and sent them back to the research team after completion of the interview.

### Analysis

The aim of the interviews was to achieve “meaning saturation,” the point at which coding subsequent interviews revealed no new thematic categories nor yielded any “further dimensions, nuances, or insights” about the thematic categories (Hennink, Kaiser, and Marconi 2017; O’Reilly and Parker 2013; Guest, Bunce, and Johnson 2006).

Two coders initially reviewed five randomly selected transcripts and identified key themes and subthemes. They then coded three randomly selected interviews at a time, iteratively refining the preliminary themes until reaching meaning saturation. This occurred at 22 interviews, at which time an additional 4 interviews were in process. All interviews were then coded using the finalized codebook; each interview was coded independently by two coders who then discussed any discrepancies. Remaining disagreements were settled by a third author. The last four interviews we coded to identify illustrative excerpts from the interviews of finalized themes. The final analysis thus includes 26 interviews. Finally, to assess how often themes were articulated, we determined which themes were mentioned by each participant (Table 1). For participants’ overall view of a PPP on a 10-point scale, we regarded scores of 1–4 as a lack of support, 5–7 as moderate support and 8–10 as strong support.

### Participant protections

To minimize distress, surrogates who had lost the patient for whom they made decisions within the previous two months were not enrolled in the study. In addition, any individuals for whom the clinicians felt the interview might be too stressful were not referred to the moderator. Participants were reminded they could choose not to answer any questions, they could end their participation at any time, and they could be referred to social work for counseling if needed. The patient for whom one of the participants was making decisions passed away after the interview had been scheduled. Because the surrogate still wanted to participate, she was included.

To protect privacy, no identifying information was collected and each respondent chose a pseudonym for the interview. The initials in the present manuscript are the initials of the chosen pseudonyms. Secondary research is limited to what is described in the consent form. The protocol, consents, surveys, and handouts were approved by the Special Studies IRB, at the National Institutes of Health with the most experience reviewing survey research.

## Results

### Participants

We enrolled 26 participants: 17 were interviewed by telephone and nine in-person. All the telephone interviews were conducted individually. Of the in-person interviews, eight participants were interviewed in groups of 2–3 persons and one was interviewed individually. The interviews lasted 90 – 120 minutes, with the exception of one phone interview which lasted 55 minutes. For the purposes of analysis, we assessed participants’ responses individually, whether they participated in an individual or a group interview. All interviews took place prior to the COVID-19 pandemic. Table 1 indicates which participants participated in individual and group interviews.

Nineteen participants self-identified as female, seven as male. Fourteen self-identified as African American, 11 as Caucasian, and one as Asian American (Table 2). Eleven of the participants were the spouse or partner of the patient, five were the patient’s children, four were the patient’s siblings, and one surrogate was the patient’s parent. Other categories of relation included the patient’s cousin, niece, the Godparents, and the siblings-in-law. Twenty had known the patient for at least 20 years, with fourteen participants who knew the patient for over 40 years. All participants were making decisions on behalf of a patient who previously had decisional capacity. Fifteen of the participants had been making decisions on behalf of a patient whose cognitive capacities suddenly declined due to an acute medical event; the remaining eleven made decisions on behalf of patients whose cognitive capacities declined slowly over time. Eighteen had made medical decisions for the patient within the past month (Table 3).

Three thematic domains emerged as the most important aspects of surrogates’ views on the idea of a PPP: Level of Support, Reasons for Lack of Support, and Surrogates’ Decisional Authority.

### Level of support

Overall, if use of a PPP increases surrogates’ predictive accuracy, 21 participants moderately or strongly supported its use. Many regarded a tool that could improve predictive accuracy as very valuable. A major benefit articulated in the interviews, and cited previously by patients, was that a PPP could improve surrogate decision-making by offering evidence of the patient’s preferences. One participant stated “Two heads are better than one, my grandma always told me. So, you listen, you learn… I think PPP is extremely helpful.” [JJ 381]. By offering supplemental information about the patient’s predicted preferences, participants saw the PPP as way to improve decision-making. Here is an example of how one respondent thought that the PPP could offer credible evidence:

“And when you look at the information that comes from the PPP it comes from information that you have vetted and its findings on the information you have; [as] opposed to when you go to Google, you’re just pulling up information that you have no idea really where it came from. It can be other people’s experiences and you can’t really base what you’re going to do on someone else’s experience.” [QL 313]

Respondents stated that use of a PPP also could help orient surrogates to considerations that should guide their decision. Rather than offer new evidence, PPP could be a helpful tool to guide the deliberation process itself and focus surrogates’ attention on their proper role. For example, RW, who had long term experience making decisions on behalf of her husband, stated:

“A plus is it gives you a road map to start off with instead of giving you a scratch piece of paper. This way you don’t have a blank, you kind of know. It kind of helps to give you a roadmap. It’s a starting point. It’s not saying it’s going to make a decision for you. It’s just saying if you need some information to help you to make your decision. So I think that would be a plus.” [RW 310]

This theme is reiterated by another experienced surrogate who made decisions for her father:

“I think I could use it, not as a standard but as some sort of touching stone for some reality base or for some objective decision making with a decision as inherently subjective… [A]t least I could say, ‘Ok well this is what the stats say.’…We have that and then I could do more research and have other facts and influences and then I could compare, but it gives me something to work from.” [PC 292]

These two surrogates articulated the possibility that a PPP could be a helpful springboard for deliberation and a way to elicit the surrogate’s own responses about what the patient would have wanted. It also could serve as a way to refocus surrogates’ attention on the fact that it is their role to try to decide on the basis of the patient’s preferences and values, rather than on their own preferences or on what they think would be best overall. As another interviewee stated:

“It’s more training people what it is they are deciding. I think that people initially don’t understand what it is they are being asked to do. So I think that anything that helps people understand what it is their responsibilities are in making these decisions if there’s a tool that will help people understand that and how they should think about making their decision then that’s useful. “ [JD 276]

This finding highlights the possibility that use of a PPP might help to improve clinician communication with surrogates about how to effectively fulfill their role (Halpern et al. 2013). The PPP might thus serve as a tool to improve surrogate decision-making by orienting the surrogates to the considerations that should be guiding their decision rather than merely adding new evidence to be incorporated in the surrogate’s belief set. Additionally, participants described a benefit not previously reported. Multiple respondents stated that surrogates often make difficult decisions on their own. By offering a tool based on input from others, the PPP could signal that surrogates are not alone. For example, one participant who was making decisions on behalf of her husband of over 60 years stated “Maybe just the fact that other people have added and put into [the PPP], it may be a little reassuring that you’re not alone in this mess and you’ll come out the other side” [IB341].

A number of respondents supported the PPP for others, but not for themselves, on the grounds that they knew the patient’s preferences. For instance, one surrogate who started making decisions on behalf of his wife following a stroke made the following distinction; “If a surrogate had no previous real discussions with the patient as to what they wanted, that might be a good crutch for them to lean on…In my case I had a good understanding of what she would want in various situations” [TH 315].

### Reasons for lack of support

Five participants did not support use of a PPP, even if it increases surrogates’ predictive accuracy. Two felt that surrogates should choose what is medically best for the patient, not what the patient would have chosen for themselves. “Another participant expressed concern that implementation of a PPP would require knowing many things about the patient: “I recognize the intent behind it, and my first thought was, well maybe if there wasn’t somebody who was really close to the patient there, they might need some help with this. But if there wasn’t someone close to the patient then how accurate is the information that you’re going to put in to find out what they might want to know?” [VD287].

Finally, one participant worried that a PPP might be biased against underrepresented groups: “My initial reaction was an algorithm, yikes! I think algorithms can be discriminatory and in other ways flawed” [OS 250].

### Surrogates’ decisional authority

The consensus among supporters and critics alike was that, if a PPP is developed, and if its use increases predictive accuracy, it should be offered to surrogates. At the same time, the overwhelming majority of respondents stated that the surrogate, and not the preferences of the patient, should decide how, if at all, a PPP is used in practice. Respondents pointed out that surrogates have the *responsibility* of making treatment decisions for decisionally-incapacitated patients. As a result, they felt that surrogates should have the *authority* to decide how to make treatment decisions, including whether to use a PPP. One surrogate who had recently started making decisions on behalf of her critically ill brother articulated:

“[T]he [PPP] kind of makes sense. And any little thing to help would help. And obviously when all is said and done, even if the result would be they should do this, it’s still the loved one’s decision. So it’s just another tool to help the person.” [JL372]

This was the overwhelming view of these experienced surrogates, even those who were most critical of the idea of a PPP. For instance, one participant who was ambivalent about the idea of a PPP due to discomfort with technology stated: “the PPP should probably still be offered to surrogates if they want it. It’s their choice” [LN 369]. Another participant, who was very enthusiastic, worried that mandating use of a PPP “could take the power out of the surrogate [and] the patient’s hands– and computerize the decision” [AH 413]. Finally, a number of respondents expressed concern that if surrogates did not have the authority to decide how to use a PPP, it might be used to deny expensive care to patients who need it.

## Discussion

If a PPP increases predictive accuracy, the majority of experienced surrogates we interviewed supported the idea of using it. This finding, together with previous findings that patients would support its use, suggests that, if a PPP increases predictive accuracy, its use would be welcome in practice.

Previous studies have found that patients endorse three primary goals with respect to surrogate decision-making: receive the treatments they want, minimize the burden on their family and loved ones, and keep their family and loved ones involved in the decision-making process (Rid et al. 2015). The present finding that experienced surrogates support the use of a PPP suggests it could offer a way to promote all three goals. Specifically, its use might increase surrogates’ predictive accuracy. If it does, its use also could decrease surrogates’ emotional burden while keeping them involved in the decision-making process. This possibility provides strong support for developing and testing a full-scale PPP to see whether its use in fact increases surrogates’ predictive accuracy in practice.

One possibility would be to use the prediction of the PPP as a “soft” default (Halpern 2018; Halpern et al. 2013). Specifically, clinicians could provide the surrogate with the treatment prediction of the PPP and suggest treating the patient accordingly, unless the surrogate objects. Thus providing the PPP prediction could help structure discussions regarding the treatment plan around an evidence-based starting point. This approach also might help to reduce the decision-making burden on surrogates who are uncertain which treatment option the patient would choose. And the use of a soft default would allow surrogates who are confident of the patient’s treatment preferences to select that option when it conflicts with the prediction of the PPP.

At the same time, the study findings highlight several important challenges. First, the findings reveal a possible fundamental disagreement between patients and surrogates regarding medical decision-making. Respondents overwhelmingly declared that ultimately, surrogates should have the *authority* to decide how to make treatment decisions, including whether to use a PPP. Consistent with this view, several respondents indicated that they would not use a PPP, even if it increases predictive accuracy, because they made decisions based on what they thought was best for the patient, not based on what they thought the patient would choose for themselves.

However, previous studies have found that a majority of patients, in contrast, prioritize making treatment decisions during periods of decisional incapacity consistent with their own preferences. In particular, most patients prefer decision-making procedures that more accurately predict their treatment preferences over methods that minimize the stress on their family and loved ones (Rid et al. 2015). These findings suggest that, if a PPP increases predictive accuracy, most patients would want it to be used, even when doing so conflicts with their surrogates’ preferences about how to make decisions on behalf of the patient.

Taken together, these findings reveal a critical need for future exploration to determine whose preferences, the patient’s or the surrogate’s, should govern surrogate decision-making. If patient preferences should be determinative, we might design advance directives to go beyond documenting patients’ treatment preferences to permit patients to indicate their preferences regarding the decision-making process itself, including whether to use a PPP. Conversely, if surrogates should have the final say, procedures should allow them to make decisions that contradict the patient’s preferences, including the patient’s preferences regarding use of a PPP. On this approach, the ultimate goal of advance care planning might be to *provide medical care* that is concordant with the patient’s treatment preferences, but not necessarily through *a deliberative process* that is determined by the patient’s process preferences.

More research is needed to determine whether we should prioritize the patient’s or the surrogate’s preferences when it comes to the decision-making process itself. In the meantime, clinicians should be aware of and take prospective steps to try to address this potential conflict. Specifically, clinicians should encourage surrogates and patients to discuss not only the patient’s treatment preferences, but also the process by which treatment decisions will be made on their behalf (Howard 2017). Does the patient want the surrogate to decide based on substituted judgment or best interests or the preferences of the surrogate? Is the surrogate willing to use that approach? If a tool such as a PPP were to become available, clinicians should discuss with their patients, and encourage their patients to discuss with their surrogates, how they would want it to be used. If the patient and surrogate cannot agree, the patient should be encouraged to consider whether they want to assign a different surrogate.

Second, a number of respondents indicated that they would not use the PPP personally because they felt confident they knew which treatments the patient wanted. This confidence is likely protective: uncertainty regarding which treatments the patient would want is a significant source of stress for surrogates. At the same time, even surrogates who have known the patient for decades often are mistaken about their charge’s treatment preferences (Fagerlin et al. 2001; Scheibehenne, Mata, and Todd 2011; Davis, Hoch, and Ragsdale 1986). Moreover, as we reviewed earlier, studies indicate that prior discussions between surrogates and patients do not increase surrogates’ predictive accuracy. Surrogates may thus feel overly confident about their predictive abilities as a result of their prior experience with the patient.

This raises an ethical dilemma that should be addressed prior to implementing a PPP. In cases where the surrogate is confident of the patient’s treatment preferences, use of a PPP has the potential to increase the chances that the patient is treated consistent with their preferences. But, its use may also undermine surrogates’ confidence and thereby increase their decision-making burden. More work is needed to assess whether we can prime surrogates for the possibility that an accurate PPP may undermine confidence that they are making the right choice. In the meantime, clinicians should be aware that confidence regarding the patient’s preferences is associated with lower surrogate burden.

Third, a PPP should be developed and implemented in a way that addresses concern that it might be used to deny expensive care to patients who need it. In addition, while only one participant raised this concern, it will be important to address the potential for algorithmic bias, especially with respect to minority populations. One worry is that the PPP may turn out to be inaccurate for certain populations if it is based primarily on the views of majority populations. As others have warned, in medicine and other contexts, algorithmic tools “reflect the biases inherent in the data used to train them.” (Char, Shah, and Magnus 2018) In order to attain predictive accuracy, a PPP might also include inputs that do not seem relevant to surrogate decision-making, such as the patient’s educational status or their zip code. Moreover, some predictors may accurately predict patient preferences, but only due to the fact that the preferences themselves are the result of unjust circumstances. For example, African Americans and white women have been found to prefer aggressive medical care at the end of life, due in part to lack of trust that medical staff will take all measures to support them (Mebane et al. 1999).

Ultimately, the efficacy of a PPP will depend not merely on its predictive accuracy, but on its social acceptability and uptake in the clinical setting. To this end, development of a PPP should include input from all groups to ensure it is not skewed in favor of majority populations. It will be critical for implementation purposes to secure the trust of all groups. One possibility in this regard would be to establish an independent board to oversee development and implementation of a PPP.

### Limitations

Our study has several important limitations that should be addressed by future research. First, our reliance on convenience sampling may have yielded an unrepresentative sample of participants. Second, we interviewed only English-speaking surrogates. Third, our participants were all recruited from urban settings and may not reflect the views of others. Fourth, most of our respondents were female, although this may mirror the surrogate population at large given gendered differences in life expectancies, caretaking responsibilities, and state laws assigning spouses as the default surrogates for patients who did not designate a surrogate (Zettel-Watson et al. 2008).

## Conclusions

Current practice frequently fails to provide care consistent with the preferences of decisionally-incapacitated patients and imposes significant emotional burden on many surrogates. The present findings that experienced surrogates support the idea of a PPP suggests its use might offer a means to address these challenges. This finding provides strong support for developing a PPP and testing its feasibility, predictive accuracy and impact in practice.

In addition, this study is the first to document a fundamental disagreement between surrogates and patients. Previous research reveals that patients believe their preferences should guide their treatment during periods of decisional incapacity. This preference is reflected in current reliance on the substituted judgment standard. However, we find that surrogates who have the responsibility to make decisions believe that they should also have the authority to decide how decisions are made. Whether to prioritize patient preferences or surrogate discretion is a foundational ethical question that should be settled prior to designing a process for implementing a PPP in practice. This question must be addressed to ensure that a PPP, or any decisional tool for that matter, is used in a way that aids surrogate decision-making, guards against further stress and trauma for families, and aligns with patient preferences and values.

## Supplementary Material

Supp 1

## Figures and Tables

**Table 1.** Themes expressed by individual participants (*N* = 26).

	Level of support	Level of support changed during interview	PPP should be offered	Surrogate should decide if use PPP	No need for PPP: I know what patient wants	No need for PPP: I use best interests	PPP might be biased	PPP might be used to deny care

PC*	Strong (8–10)		✓	✓				
WS*			✓	✓				
RK†			✓	✓				
MS*			✓	✓				
MJB*			✓	✓				
DG*			✓	✓				
JL†			✓	✓				
JEJ*			✓	✓				
TH*			✓	✓	✓			✓
IB*			✓	✓	✓			
VD†	Moderate (5–7)					✓		
QL*		[+]	✓	✓		✓		
RM*			✓	✓		✓		
JN†		[−]	✓	✓				
BD†				✓				✓
RW*			✓	✓				
JD*			✓	✓	✓			
JG†				✓				✓
LN*		[−]	✓	✓				
MM*				✓	✓			✓
HB*			✓	✓		✓		
WG*	Lack of Support (1–4)		✓	✓				
AH†			✓	✓	✓			
MN*			✓	✓		✓		
OW*			✓	✓				
OS†						✓	✓	✓
Totals		3	21	24	5	6	1	5

* connotes participation in individual interview.

† connotes participation in group interview.

**Table 2.** Participant characteristics (*N* = 26).

Characteristic	*N*

**Sex**	
Female	19
Male	7
**Age**	
30–50	9
51–70	13
71–90	3
Missing	1
**Race**	
African-American	14
White	11
Asian	1
**Ethnicity**	
Hispanic	0
non-Hispanic	26
**Education level**	
High school	6
College	8
Graduate School	10
Missing	2

**Table 3.** Surrogate characteristics (*N* = 26).

Relationship to the patient	

Spouse	11
Parent	5
Sibling	4
Other	6
**Years knew patient**	
0–19	6
20+	20
**Treatment decisions made as surrogate**	
1–3	4
4–10	9
11+	12
Missing	1
**Made decision(s) in past month?**	
Yes	18
No	7
Missing	1
