# Supplementary material for: Surrogate Perspectives on Patient Preference Predictors: Good Idea, but I Should Decide How They Are Used
Source: AJOB Empir Bioeth. Author manuscript; Available in PMC 2023 Apr 1. (PMC9761590; doi:10.1080/23294515.2022.2040643)
Supplement: Supp 1 [file NIHMS1834959-supplement-Supp_1.docx]

**Supplemental Digital Content**

**Supplemental Digital Content 1: Interview Guide**

**Introduction**

Moderator introduces themselves and shares the interview ground rules.

Voluntary and confidential

If you are feeling overwhelmed or distressed by the conversation, feel free to step out of the room.

You can stop participating at any point in the session.

Tape recorder and observers

There are no wrong or right answers, I am just interested in hearing your point of view and personal experiences.

One speaker at a time. And I want to make sure everyone has a roughly equal chance to speak.

We are going to be talking about making medical decisions for patients who are not able to make their own decisions. For example, when someone is in a car accident and is unconscious, or they have a stroke and cannot communicate, a family member or loved one must make medical decisions for them. We call the person who makes these decisions the patient’s surrogate decision maker. This is something millions of people have personally experienced including each of you.

This project has two goals. First, we want to learn what it was like for each of you to make medical decisions for a loved one. Second, we are interested in what you think about a new method that could be used to help surrogates make medical decisions for others.

I am not a doctor. My expertise is in asking questions and listening to you. For this project I am most interested in your views and experiences, rather than the medical details of the patient and what they were facing.

Participants introduce themselves with first name and the first name of the person for whom they were making decisions.

**Experiences as a Surrogate**

Let’s start by having you write down two words that would help me to understand your experience as a surrogate decision maker. When you think about what it was like to make medical decisions for someone else, what one or two words come to mind? (If you have had more than one experience as a surrogate, please choose just one to talk about today – for most people this should be the most recent. But if you have had more than one experience within the last three years you might want to talk about the experience that was most significant for you.)

What words did you write?

What was it like for you to make medical decisions for a loved one?

Did you involve other family members or close friends in the decision, or were you deciding primarily on your own?

How did you understand your role as a surrogate decision maker? What was your goal in making decisions? How were you supposed to decide which decisions to make? What did you consider a correct decision?

[Handout A] This handout gives you a few chances to tell me about your personal experience as a surrogate with some ratings on a zero to 10 scale.

First, please rate the level of stress you experienced using a zero to 10 scale where zero means you did not experience any stress, and 10 means you were completely stressed out.

Next, please rate the level of confidence you had at the time that you made the right decisions – please use a zero to 10 scale where zero means you did not have any confidence, and 10 means you were completely confident.

Did anyone of you have a chance to talk to the patient after you had to act as their surrogate? Did this change how you thought about the decision that you made?

Then, rate the level of information you had to make the decisions. This time use a slightly different scale from zero to 15 where zero means no information at all, 10 means all of the information you needed or wanted, and numbers above 10 represent information overload. Eleven means you were given a bit too much information and 15 means you were completely overloaded with information.

Tell me about the stress you experienced. What was most stressful about the situation? Did you find the experience positive or rewarding in any way? What was rewarding?

Tell me about the information you had at the time. What did you know and what didn’t you know that would have been important to you in making your decision? How did you get this information?

At the time you were making these decisions, which was the bigger priority for you: making sure that the patient gets the treatment he or she wanted or making sure the patient was receiving treatment that was in his or her best interest? Or what the family thought would be best for the patient?

I now want to take a moment to check how people are doing. For some of you it must be very difficult to talk about your experiences. Before we move on, I want to remind you that you are free to take a break from the conversation or end your participation at any time.

**Introduction of the PPP**

[Handout B – Description of the PPP]. Please read the statement and give the idea a rating on the zero to 10 scale. Also please write in the margins any comments or questions you have about the handout.

Many patients cannot make their own decisions. For example, people in car accidents sometimes need treatment when they are unconscious. In these cases, doctors do what is best for the patient. However, sometimes it is not clear what would be best for the patient. In these cases, the doctors and family members or loved ones must decide how the patient is treated.

Medical researchers are thinking about developing a new computer program they call the Patient Preference Predictor or PPP for short. The goal of the program would be to predict what treatment patients want when they are not able to make their own decisions in the most common and difficult cases. It works by the doctors entering information about the patient, such as their disease or injury, their age, their gender, their race, religious background, or education level.

The program predicts how the patient would want to be treated based on data about how patients who share the same characteristics want to be treated in similar circumstances. The PPP predictions also come with a measure of confidence about how likely it is that the family member would want a certain treatment. For example, based on the information entered about the patient, the PPP program might predict that there is a 90% chance that the patient would want have a certain treatment.

The surrogate could take this information into account when making a decision for the patient. Studies suggest that the PPP might help surrogates to predict patients’ preferences more accurately. We are interested in what experienced surrogates like you think about this possible approach. We want to know to what extent you think developing such a program would be a good or bad idea

What is your reaction to this handout?

What do you think of the idea? What ratings did you give?

Please give the description a rating on a zero to 10 scale for how well and clearly it explains what a Patient Preference Predictor is.

Now give yourself a letter grade to let me know how well you think you understand what the Patient Preference Predictor really is. Poll for grades.

Let me have the As and Bs hold out for a moment and have the Cs, Ds, and Fs answer: What is the main idea here? How does this work? OK, Now the As and Bs can answer: What is the key idea here?

**Evaluation of the PPP**

In what ways would the Patient Preference Predictor have been a plus or a minus for you when you were acting as a surrogate?

[On easel] What do you see as the pluses and minuses of the Patient Preference Predictor?

[Handout C]: Let me capture where we are in the discussion with this handout that has a few more questions about the Patient Preference Predictor (PPP). Starting with the top of the page…

If the PPP had existed when you were acting as a surrogate decision maker, please check the box to tell me which of the following choices comes closest to your preference:

I would not want to know about it.

I would want to know about it but would not want to use it.

I would want to know about it and may have chosen to use it if the situation was appropriate

I would want to use it to know what it predicted for my family member’s preferences.

How did you answer the question?

Which box did you check?

Help me understand your reaction to this question.

At the bottom of the handout, there are three modes of decision-making in the event of incapacity – Please put a check by the one you think would be best from the point of view of the surrogate decision-maker.

The surrogate and the patient’s doctor first discuss the patient’s situation and possible treatment options, then:

1. The surrogate makes the final decision for treatment (If they wish, the surrogate may choose to consult the Patient Preference Predictor or do what the doctor suggests.)

2. The doctor makes the final decision for treatment. (If they wish, the doctor may choose to consult the Patient Preference Predictor or do what the surrogate suggests.)

3. The doctor and the surrogate make the final decision together. (If they wish, they may choose to consult the Patient Preference Predictor to help them decide.

4. The patient is treated based on the PPP prediction unless the doctor or the family disagrees. Here the PPP acts as the default course of treatment but either the doctor or family can object if they disagree with the PPP recommendation.

What do you see as the positives and negatives for each of these options?

Which approach did you think was best from the surrogate’s point of view?

Would you see it as a plus or a minus if the Patient Preference Predictor is consulted in options 1 and 2?

**The Patient Point of View**:

So let’s flip the roles: What if you were answering these questions from the patient’s point of view? Realizing you could be the patient sometime in the future, how does this change your view of these options?

If you were in a bad car accident or had a stroke that left you unconscious. And a decision has to be made of whether to perform a surgery on you. The surgery has risks and potential benefits. It may increase the chances that you regain your normal abilities, like walking and talking. But, it may not work and may simply increase your pain. It is unclear whether it is best for you to have the surgery. In this case, how would you like the decision to be made of whether you have the surgery?

**Accuracy and Value**

How accurate do you think the Patient Preference Predictor would be?

How accurate would the Patient Preference Predictor have to be for you to feel comfortable using it: 90% accurate? 85% accurate? 80% accurate? 75% accurate?

Suppose a Patient Preference Predictor could be developed that would predict your loved one’s preferences with 85% accuracy, but it would be expensive. How much would you be willing to pay of your own money?

Do you think that the patient should have paid for it if they want you to use it?

Do you think surrogates should have the option to pay for it if they want to use it?

How much do you think society should be willing to pay to develop it?

Suppose a Patient Preference Predictor could be developed that would predict your loved one’s preferences with 85% accuracy, but you think that the prediction is wrong in your family member’s case. What would you do? Would you trust your view of your family member’s preference or the Patient Preference Prediction in deciding what treatment you ultimately choose for the patient?

Suppose a Patient Preference Predictor is developed and it predicts that the patient would have wanted treatment in these particular circumstances, however the patient’s doctor does not think that treatment is in the patient’s medical interests. :How would you decide on the patient’s behalf at this point – should the doctor’s judgment or the Patient Preference Predictor be followed?

**Supplemental Digital Content 2: Quantitative Survey: Pre-Interview**

1. How long have you known the patient? _______ years.

2. What is your relationship to the patient?

1. Patient’s Spouse/partner
2. Patient’s child
3. Patient’s sibling
4. Patient’s parent
5. Other relative (please describe): _______________________
6. Other (please describe): ______________________

3. Would you say that:

1. The patient’s ability to make decisions declined slowly over time
2. The patient’s ability to make decisions declined suddenly.

4. Prior to becoming the surrogate decision-maker, how familiar were you with the patient’s medical treatment preferences:

1. Extremely familiar
2. Moderately familiar
3. A little familiar
4. Not familiar at all

5. Approximately how many treatment decisions have you made for the patient?

a. 1-3

b. 4-10

c. More than 10

6. Which option best describes your situation

a. I am currently making treatment decisions for the patient

b. I have made treatment decisions in the past month

c. I made treatment decisions in the past year

d. I made treatment decisions more than a year ago

7. When it comes to making important treatment decisions for the patient, would you say that:

1. I usually made (make) the important decisions
2. The patient’s physician usually made (makes) the important decisions
3. The patient’s physician and I usually made (make) the important decisions together
4. Other: _______________________________________

8. Who would you say SHOULD make the important treatment decisions for the patient?

1. I should make the important decisions
2. The patient’s physician should make the important decisions
3. The patient’s physician and I should make the important decisions together
4. Other: ____________________________________________________

9. In general, how often do you think DOCTORS know what treatment is medically best for patients who are unable to make their own decisions?

1. Doctors always know what treatment is medically best for the patient (100%)
2. Doctors usually know what treatment is medically best for the patient
3. Doctors know about half the time what treatment is medically best for the patient (50%)
4. Doctors rarely know what treatment is medically best for the patient
5. Doctors never know what treatment is medically best for the patient (0%)

10. In general, how often do you think SURROGATES are able to determine which treatment decision the patient would have made for themselves?

a. Always

b. Frequently

c. About half the time (50%)

d. Rarely

e. Never

11. When making treatment decisions for patients who can’t make their own decisions, how important are the following things?

| *How important is…?* | Extremely important | Moderately important | Just a little important | Not important at all |
| --- | --- | --- | --- | --- |
| Making the treatment decision the patient would have made for themselves | ☐ | ☐ | ☐ | ☐ |
| Making the decision that is best for the patient medically, regardless of what the patient would have chosen for themselves | ☐ | ☐ | ☐ | ☐ |
| Making the decision that is the least stressful for the family | ☐ | ☐ | ☐ | ☐ |
| Making the decision that the family thinks is best | ☐ | ☐ | ☐ | ☐ |

12. How confident are you that you made the treatment decisions the patient would have made for themselves?

1. Extremely confident
2. Moderately confident
3. A little confident
4. Not confident at all

13. How confident are you that you made the treatment decisions that were medically best for the patient?

1. Extremely confident
2. Moderately confident
3. A little confident
4. Not confident at all

**Supplemental Digital Content 3: Quantitative Survey: Post-Interview**

1. In general, how often do you think DOCTORS know what treatment is medically best for patients who are unable to make their own decisions?

1. Doctors always know what treatment is medically best for the patient (100%)
2. Doctors usually know what treatment is medically best for the patient
3. Doctors know about half the time what treatment is medically best for the patient (50%)
4. Doctors rarely know what treatment is medically best for the patient
5. Doctors never know what treatment is medically best for the patient (0%)

2. In general, how often do you think SURROGATES are able to determine which treatment decision the patient would have made for themselves?

a. Always

b. Frequently

c. About half the time (50%)

d. Rarely

e. Never

3. What impact do you think use of the patient preference predictor [PPP] would have on surrogates’ ability to determine which decision the patient would have made for themselves? Would you say that:

1. Use of the PPP would **increase** surrogates’ ability to determine which decision the patient would have made for themselves
2. Use of the PPP would **not affect** surrogates’ ability to determine which decision the patient would have made for themselves
3. Use of the PPP would **decrease** surrogates’ ability to determine which decision the patient would have made for themselves

4. What impact do you think use of the patient preference predictor [PPP] would have on surrogates’ level of stress? Would you say that:

1. Use of the PPP would **increase** surrogates’ stress
2. Use of the PPP would **not affect** surrogates’ stress
3. Use of the PPP would **decrease** surrogates’ stress

5. Do you think that the PPP should be offered to surrogates as a method to help them to make treatment decisions?

1. The PPP definitely should be offered to surrogates
2. The PPP probably should be offered to surrogates
3. The PPP probably should not be offered to surrogates
4. The PPP definitely should not be offered to surrogates

6. If the PPP had been offered to you, would you have used it when you were making treatment decisions for the patient?

1. I definitely would have used it
2. I probably would have used it
3. I probably would not have used it
4. I definitely would not have used it

7. Do you have any further thoughts or comments for us?

**Supplemental Digital Content 4**

**Handout A:**

## This Handout gives you a few chances to tell me about your personal experience as a surrogate with some ratings on a zero to 10 scale.

### How much stress did you experience as a result of the patient being ill?

0 1 2 3 4 5 6 7 8 9 10

Felt completely stressed out

Did not experience any stress

### How much stress did you experience as a result of making the medical decision for the patient [reminder: please focus on one significant decision that you made]?

0 1 2 3 4 5 6 7 8 9 10

Felt completely stressed out

Did not experience any stress

### How confident are you that you made the decision the patient would have made for themselves if they had been able to make their own decisions?

0 1 2 3 4 5 6 7 8 9 10

I was completely confident

I had no confidence

1. What did you think about the level of information that was provided to you to help you make the decision?

0 1 2 3 4 5 6 7 8 9 10 11 12 13 14 15

I was provided too much information; I was completely overloaded

I was provided all the information I needed and wanted

I was provided no information

Did not experience any stress

**Supplemental Digital Content 5: Handout B**

*Please read the following description and give the idea a rating on the zero to 10 scale. Also please write in the margins any comments or questions you have about the PPP.*

Many patients cannot make their own treatment decisions. For example, people in car accidents sometimes need treatment when they are unconscious. Or patients who have a stroke sometimes cannot communicate. Doctors do what is best medically for these patients. However, in some cases, it is not clear what is medically best for the patient.

 In these cases, the doctors and the patient’s surrogate must make the treatment decisions for the patient. These decisions can be difficult, especially when it is not clear how the patient would want to be treated. Medical researchers are developing a new method, called the patient preference predictor (PPP for short) to try to help doctors and surrogates make these decisions.

 The PPP is designed to help surrogates by predicting how the patient would want to be treated. It does this based on how other patients want to be treated when facing similar circumstances.  To develop a PPP, researchers would ask thousands of people how they want to be treated in different situations. The researchers would use their answers to develop the PPP.

Once the PPP is developed, doctors would enter into a computer the patient’s condition and their treatment options. The surrogate would then provide information about the patient, such as their age, gender, how religious they are, their prior level of health and physical activity, and whether they tend to be risk averse or risk takers. The PPP would predict how the patient would want to be treated based on how people who share the same characteristics want to be treated in similar circumstances.

For example, the PPP might predict, based on the patient’s situation and personal characteristics, that there is a high chance the patient would want surgery. In another case, the PPP might predict that there is a high chance the patient would not want dialysis. The surrogate could take this information into account when deciding how the patient should be treated.

*What do you think about the idea of a PPP?*

0 1 2 3 4 5 6 7 8 9 10

Not helpful at all

Extremely helpful

**Supplemental Digital Content 6: Handout C**

1. If a patient preference predictor [PPP] had been available, how useful do you think it would it have been when you were making the treatment decision for the patient?
2. Extremely useful
3. Moderately useful
4. A little useful
5. Not useful at all
6. If a PPP had been available, how do you think its use would have affected your stress level?
7. It would have made my decision-making much more stressful
8. It would have made my decision-making somewhat more stressful
9. It would have not had any impact on my stress level
10. It would have made my decision-making somewhat less stressful
11. It would have made my decision-making much less stressful
12. Do you think that the PPP should be offered to surrogates when they are making treatment decisions for their loved ones?

1. The PPP definitely should be offered to surrogates
2. The PPP probably should be offered to surrogates
3. The PPP probably should not be offered to surrogates
4. The PPP definitely should not be offered to surrogates

**Supplemental Digital Content 7: Handout D**

Now consider a case in which you are the patient and you are unable to make your own medical decisions:

| 1. Overall, how often do you think your family would make the treatment decisions that you would have made for yourself? | |
| --- | --- |
| ⬛⬜ | Always (100%) |
| ⬛⬜ | Frequently |
| ⬛⬜ | About half the time (50%) |
| ⬛⬜ | Rarely |

| 2. How much stress do you think your family would experience as the result of making treatment decisions for you? | |
| --- | --- |
| ⬛⬜ | A lot of stress |
| ⬛⬜ | A moderate amount of stress |
| ⬛⬜ | Just a little stress |
| ⬛⬜ | No stress at all |

| 3. If the patient preference predictor [PPP] were available, how would you want your doctors to use it? |
| --- |

⬜ Not at all: My family should help the doctors make decisions without any information on which treatments people like me want.

⬜ Give my family the option of using it: The doctors should let my family decide whether they use the PPP.

⬜ Use it as an added information: The doctors should give my family the information on which treatments people like me want, and let my family decide how I am treated.

⬜ Use it as the default choice: The doctors should give me the treatments people like me want unless my family believes I would want some other treatment.
